# Phase diagrams of PEG_1000,1500,2000,4000,6000_ + lithium citrate + water ATPSs, and the partitioning of salbutamol at *T* = 298.15 K

**DOI:** 10.1038/s41598-023-28046-9

**Published:** 2023-01-19

**Authors:** Ebrahim Nemati-Kande, Zolfa Azizi, Marziyeh Mokarizadeh

**Affiliations:** Department of Physical Chemistry, Chemistry Faculty, Urmia University, Urmia, Iran

**Keywords:** Thermodynamics, Physical chemistry

## Abstract

Salbutamol is a drug used to treat the pulmonary diseases by ameliorate the medium and large airways in the lungs. Partitioning of salbutamol drug on the aqueous two-phase systems (ATPSs) of PEG_1000,1500,2000,4000,6000_ + trilithium citrate + water was determined at *T* = 298.15 K. The effect of molecular mass of polymer (MMP) on the binodal and tie-line compositions were studied. Results showed that the biphasic area was extended as the MMP was increased. The salting-out ability were quantified using the Setschenow model, and the binodal curves were modeled by a nonlinear 3-parameter equation. Furthermore, electrolyte Wilson along with the osmotic virial models have adequately been implemented to fit the tie-line compositions. Also, the studied ATPSs were implemented to study the partitioning of salbutamol drug on the salt-affluent and polymer-affluent phases. It is observed that, ATPSs of PEG_1000_ is premium to extract the salbutamol to the polymer-affluent phase, where, the ATPSs of PEG_6000_ is more favorable to extract the drug to the salt-affluent phase.

## Introduction

Considering the significant capabilities of aqueous two-phase systems (ATPSs) in chemical and especially biological studies, they have been used frequently in partitioning, separation and purification of the proteins^1^, biomolecules^2^, chemical, and pharmaceutical materials^3^. ATPSs can be composed by mixing appropriate amounts of aqueous solution of two polymers, or one polymer and a salt, which has been separated into two-phases. Due to numerous features such as being economical^4^, easy to scale up^5^, selectivity^6^, fast separation ability^7^, and high resolution^8^, ATPSs obtained from the mixing of a hydrophilic polymer and a kosmotropic salts are more favorable than the aqueous solutions of two polymers.

The most favorable polymer used in ATPS studies is the hydrophobic polyethylene glycol (PEG). Generally, PEG and Dextran are used in small scale separation; however, PEG + salt systems are more desirable in industrial scales^9^. There are many scientific reports on the ATPSs of polymer-salt-water systems, which affiliate to the PEG and inorganic salts such as, phosphates^10^, sulfates^11^, or nitrates^12^. However, citrate salts are proper substitute to inorganic salts in the extraction of biological, and food materials, due to low toxicity and high solubility^13,14^.

ATPS of PEG_1000,4000,6000_ + cesium chloride (CsCl) + water at *T* = 288.15 and 308.15 K were studied by Wang et al.^15^ They found that, an increase in the temperature and molecular mass of polymer (MMP), enhanced the liquid–liquid two-phase formation tendency^15^. Barani et al.^16^ studied the efficiency of PEG_1000_ and PEG_6000_ in the presence of C_4_H_4_K_2_O_6_ at various pH and temperatures, and the biphasic diagrams and tie-line compositions were determined experimentally. Graber et al.^17^ studied the ATPSs of PEG_2000,6000,10000_ + NaNO_3_ + water at the room temperature, and found that, the two-phase area was extended with raising the MMP. In a similar work, Hey and colleagues^18^ used PEG_8000_ in combination with different electrolytes such as hydroxide, carbonate, sulphate, hydrogen phosphate, and phosphate anions with Na, Mg, and Zn cations to attain the thermodynamic equilibria, and to study the salting-out capability.

The solvation of PEG in aqueous solution is principally controlled by intermolecular hydrogen-bonding of solute–solvent type. Nemethy et al.^19^ studied the distribution of ethylene glycol molecules by examining the relationship between the temperature and some physical properties, and concluded that, the three-dimensional structures of the intermolecular solute–solvent hydrogen-bonding in the liquid mixture is principally possible^19^. In another work, Kaulgud^20^ and coworkers showed that the intramolecular hydrogen-bonding is independent of temperature, whereas inversely, the number of intermolecular hydrogen-bonds decreases significantly with temperature enhancement, which indicates the breakdown of the intermolecular hydrogen bonds with increasing the temperature^20^.

Zafarani-Moattar et al.^21^ measured phase equilibrium of PEG + K_3_Cit + water at *T* = 293.15 to 318.15 K. They also, studied the two-phase forming capability of this system, and reported that, as the temperature enhanced, the salting-out capability of the ATPS were increased. Shahrokhi et al.^22^ investigated the biphasic system of PEG_1500_ + ZnSO_4_ + water at the room temperature. Ketabi and coworkers^23^ reported the binodal curves of the system including PEG_3000_ + tripotassium citrate + water at various pHs. The phase equilibria of the ternary systems of PEG_600,1500,3000,6000_ + sodium and potassium formate + water at different temperatures and pHs was studied experimentally by Pirdashti et al.^24^ In a similar work, they studied the two-phase equilibria for PVP + potassium hydrogen phosphate + water ATPS at *T* = 298.15 K and pH = 7.54, 8.05, and 9.47. They applied a nonlinear equation^25^ to fit the experimental binodal data. The tie-line concentration have also been fitted to the by Othmer-Tobias and Bancroft models^26^.

Polymer-salt ATPSs are accomplished for partitioning of chemical and pharmaceutical materials. Polymers with high MMP, are appropriate to separate the biphasic systems^27^, whereas, several factors such as MMP, temperature, concentration of components, and the surface, shape and compositions of the material are affecting the partitioning phenomenon^28^. Shiran and coworkers^29^ studied the effect of polymer and salt mass fraction, the cation types, temperature, and volume factor on the curcumin partitioning by using salt + polymer + water ATPS. The results for phase equilibrium of ATPSs composed of PEG_4000,6000,8000_ in the presence of potassium citrate and potassium sodium tartrate have been reported by Wysoczanska and coworkers^30^. Wang et al.^10^ reported the effect of temperature, MMP, and the concentration of PEG and Na_2_SO_4_ on the partition of molybdenum. Kalaivani and Regupathi^31^ studied the partitioning of α-lactalbumin on the ATPSs consist of different MMP of PEG (i.e., MMP = 1000, 2000 3000, 4000, and 6000) and citrate salts such as C_6_H1_7_N_3_O_7_, C_6_H_5_K_3_O_7_, Li_3_C_6_H_5_O_7_, and Na_3_C_6_H_5_O_7_^31^. Iyyaswami et al.^32^ used the aqueous solutions of PEG_4000,6000,8000_ and lithium citrate to study the phase equilibria at *T* = 298.15 K, and used the studied ATPSs in the partitioning of the proteins of fish industry affluent. Just recently, our group^33^ investigated the phase equilibrium of PEG_2000_ + trilithium citrate + water ATPSs at several temperatures, and the results of the curcumin partitioning have also been presented.

Moreover, polyethylene glycol is a water-soluble polymer, and due to flexibility, non-toxicity, low cost, and environmentally friendly properties is often used to study liquid–liquid extraction in the chemical and food industries. Also, due to the high-toxicity of sulfates, phosphates, nitrates^34,35^, and carbonate salts, they can be replaced with non-toxic citrate salts. The half-lethal dose factor (LD_50_) for lithium carbonate, lithium sulfate, and lithium citrate salts were reported to be 1590, 1959, and 2993, respectively^13^. The more the amount of the LD_50_ factor, the lower the toxicity of the salt; so, the lithium citrate is less-toxic than carbonate and sulfate salts. Therefore, the ATPSs composed of PEG and lithium citrate is a non-toxic and environmentally friendly medium for extraction and purification of different chemicals and especially drugs, which was encouraged our group to study the ATPSs of PEG + lithium citrate + water systems.

Salbutamol is a drug with commercial name of albuterol and chemical formula of C_13_H_21_NO_3_ was first discovered in the laboratory by David Jack in 1966; afterwards, became available to public in 1969 in UK. Salbutamol generally used as treatment of pulmonary diseases^36^, and control of the blood potassium level by increasing the transfer of potassium ions to the intracellular space^37^.

In this work, the partitioning of salbutamol in ATPSs of PEG_1000,1500,2000,4000,6000_ + lithium citrate + water has been investigated, and the effect of MMP on the binodal data and tie-line composition were studied. Also, a 3-parameter relation was utilized to fit the experimental binodal composition. The salting-out ability was quantified using the Setschenow-type model^18^. In addition, Osmotic virial^38^ and the e-Wilson^39^ thermodynamic models were applied to correlate the phase-equilibrium conditions.

## Methods

### Materials

Trilithium citrate and polyethylene glycol (PEG), with the MMP of 1000, 1500, 2000, 4000 and 6000 g mol^−1^ were purchased from Merck. Similar to the previous work^40^, trilithium citrate was heated in a laboratory oven at T = 378.15 K under Ar atmosphere for about two hours to achieve the anhydrous form. Moreover, double distilled deionized water with the conductivity of 0.7 μ*S*·cm^−1^ was used in all experiments. The chemical names, purifications methods, and other information of the utilized materials were summarized in Table 1.

**Table 1:** Chemicals used in this work.

Chemical	Purification method	Chemical formula	Source	Purity^a^ (mass fraction)	M_w_ (g·mol^−1^)	CAS no
Trilithium citrate	Thermal	Li_3_C_6_H_5_O_7_	Merck (Germany)	98%	209.923	6080-58-6
PEG_1000_	–	H(OCH_2_CH_2_)_n_OH	Merck (Germany)	> 99%	~ 1000	25322-68-3
PEG_1500_	–	H(OCH_2_CH_2_)_n_OH	Merck (Germany)	> 99%	~ 1500	25322-68-3
PEG_2000_	–	H(OCH_2_CH_2_)_n_OH	Merck (Germany)	> 99%	~ 2000	25322-68-3
PEG_4000_	–	H(OCH_2_CH_2_)_n_OH	Merck (Germany)	> 99%	~ 4000	25322-68-3
PEG_6000_	–	H(OCH_2_CH_2_)_n_OH	Merck (Germany)	> 99%	~ 6000	25322-68-3
Salbutamol	–	C_13_H_21_NO_3_	Exir pharmaceutical Co. Boroujerd(Iran)	≥ 98%	239.31	18559-94-9
Water	Double-distilled deionized	H2O^b^	Ghatreh (Iran)	> 99.9%	18.015	7732-18-5

^a^Purity as provided by suppliers.

^b^Double-distilled deionized water with the conductivity of 0.7 μ*S*·cm^−1^ was used.

### Apparatus and procedure

The experimental procedure contains three main parts, i.e., measurement of the binodal curves from the cloud-point titration, estimation of the tie-line compositions, and finally, study of the partitioning of the salbutamol drug in the proposed tie-lines.

The apparatus used to measure the binodal curves is like our previous works^33,41^. A glass-cell with the volume of ~ 100 ml was inserted in a one-liter volumetric flask, and the temperature of the double-wall glass container was controlled by circulation the thermostated water between the walls of the container. The aqueous PEG stock solutions (~ 40 to 60% *w*/*w*) and trilithium citrate (~ 35% *w*/*w*) were prepared for binodal measurements. About 2–4 g of PEG or salt stock solution were used to fill in the double-wall glass cell, while, the solution was mixed by a magnetic stirrer. The titrant solution was added into the cell dropwise, by a normal syringe, until the appearance of the turbidity of the solution. This determine that the solution is in the two-phase region of the LLE phase-diagram. Water droplets, with a mass of ~ 3.5 × 10^−4^ g per drop, then was added dropwise until the solution swiftly became transparent, which locate a point on the binodal curve. This procedure was repeated to measure more points on the binodal curve, and at each step, using an analytical balance (A&D Company, model No. GH-320, Japan with the precision of 1 × 10^−4^ g) the mass of the syringes containing the titrant (i.e., polymer or salt solution), and water were determined, and the binodal curve compositions were calculated. During this process, the water circulator-thermostat (Fanavaran Sahand Azar Co., model No.12 uc5000s, Iran) was set at *T* = 298.15 K. The temperature of the double-wall glass cell was further controlled using a normal thermometer with the precision of 0.1 K. The repetitive experiments were continued for 5 times for each desired mass of the polymer, and the mean standard uncertainty (*u*) of the mass fractions (*w*) was found to be *u*(*w*) = 0.0009.

The tie-line samples were prepared by mixing appropriate amounts of PEG stock solution (40 to 60% *w*/*w*), and salt solution (~ 35% *w*/*w*), and double-distilled deionized water in the closed glass containers to obtain the total solution of ~ 30 g. The PEG total mass fraction in the stock solutions was fixed at ~ 15% *w*/*w* for PEG_1000–4000_, and 25% *w*/*w* for PEG_6000_. Also, the mass fractions of the salt were selected such that the final solution be in the biphasic region. The thermostat was set to the temperature of *T* = 298.15 K, the closed containers were immersed in the thermostated water for at least 72 h, and the mixture was allowed to achieve the thermodynamic equilibrium. Then, the polymer-affluent phase was separated by a long-needle syringe from the salt-affluent phase. Also, the remaining solution of the bottom-phase was depleted into separate vessels, and the mass of the top and bottom phases were measured to calculate the composition of each component in each phase from the gravimetric analysis method proposed in our previous work^13^.

Following our previous work^13^, the mass-fractions (*w*) of the components in both phases were determined by numerical solution of the subsequent set of equations:

1 $$w_{1}^{{{\text{top}}}} = f(w_{2}^{{{\text{top}}}} )$$

2 $$w_{1}^{{{\text{bot}}}} = f(w_{2}^{{{\text{bot}}}} )$$

3 $$w_{1}^{{{\text{top}}}} = \frac{{w_{1}^{{{\text{bot}}}} }}{{w_{{{\text{tot}}}}^{{{\text{top}}}} }}(w_{1}^{{{\text{mix}}}} - w_{1}^{{{\text{bot}}}} ) + w_{1}^{{{\text{mix}}}}$$

4 $$w_{2}^{{{\text{top}}}} = \frac{{w_{2}^{{{\text{bot}}}} }}{{w_{{{\text{tot}}}}^{{{\text{top}}}} }}(w_{2}^{{{\text{mix}}}} - w_{2}^{{{\text{bot}}}} ) + w_{2}^{{{\text{mix}}}}$$

5 $$w_{1}^{{{\text{top}}}} + w_{2}^{{{\text{top}}}} + w_{3}^{{{\text{top}}}} = 1$$

6 $$w_{1}^{{{\text{bot}}}} + w_{2}^{{{\text{bot}}}} + w_{3}^{{{\text{bot}}}} = 1$$

In these relations, *w*_1_, *w*_2_, and *w*_3_ are the mass fractions of PEG, salt, and water, respectively. Also, “*top*”, “*bot*”, and “*mix*” superscripts referred to the polymer-affluent, salt affluent, and the stock solutions of the primary solution, respectively. Equations (1) and (2) are the mathematical representation of the binodal curves, which is presented for polymer-affluent region (i.e., Eq. (1)), and the salt-affluent region (i.e., Eq. (2)). Moreover, Eqs. (3) and (4) are derived from the thermodynamical lever-rule, and the remaining equations shows the mass balance of the coexisting phases. For each stock solution, the tie-line compositions repeated at least 3 times, and the reported data are the arithmetic average of the obtained results. The mean of the standard uncertainty (*u*) for the calculated mass fractions (*w*) of the tie-lines was estimated to be *u*(*w*) = 0.0014. The separated top and bottom phase samples were kept in closed glass containers to be used as blanks in determination of the concentration of salbutamol.

The designed tie-lines were also used to study the salbutamol partitioning in two-phases of the PEG + trilithium citrate + water ATPSs. For this purpose, the tie-lines with concentrations equal to the previous step were prepared and ~ 0.003 g of salbutamol was added into each sample. The samples then were settled in the thermostated bath at the temperature of *T* = 298.15 K for at least 48 h, and then, the two phases were separated. Consequently, the UV–visible absorption spectrophotometry was implemented to determine the salbutamol concentration in top and bottom phases. Different chemicals with different concentrations of polymer or salt have different wavelengths in UV–visible spectra. So, different standard calibration curves of salbutamol (0–10 ppm) were prepared for each drug-containing-phase using the remaining values of each individual phase samples kept from the previous section. To measure the absorbance of salbutamol in each sample, a quartz cell with the cell-length of 1 cm was used in UV–visible spectroscopy (Biochrom company, model no. Libra S12, U.K). The measurement was made at *λ*_max_ = 428 nm. Each sample was diluted to be in the range of the calibrations curve, and the polymer and salt affluent salbutamol-free solutions, remaining from previous step, were diluted with the same dilution factor, and used to prepare the blank solution.

The partitioning coefficient, *D*, the extraction efficiency percent, *E*%, and the separation coefficient, *R*, were calculated from the following equations:

7 $$D = {{C_{top} } \mathord{\left/ {\vphantom {{C_{top} } {C_{bot} }}} \right. \kern-0pt} {C_{bot} }}$$

8 $$E\% = {{100C_{top} m_{top} } \mathord{\left/ {\vphantom {{100C_{top} m_{top} } {\left( {C_{top} m_{top} + C_{bot} m_{bot} } \right)}}} \right. \kern-0pt} {\left( {C_{top} m_{top} + C_{bot} m_{bot} } \right)}}$$

9 $$R = {{m_{top} } \mathord{\left/ {\vphantom {{m_{top} } {m_{bot} }}} \right. \kern-0pt} {m_{bot} }}$$

where *C* refers to the salbutamol concentration (ppm), and *m* is the total mass of the salbutamol-free polymer- and salt-affluent phases.

## Results and discussion

### The measured binodal and tie-line concentration

**Figure 1:**
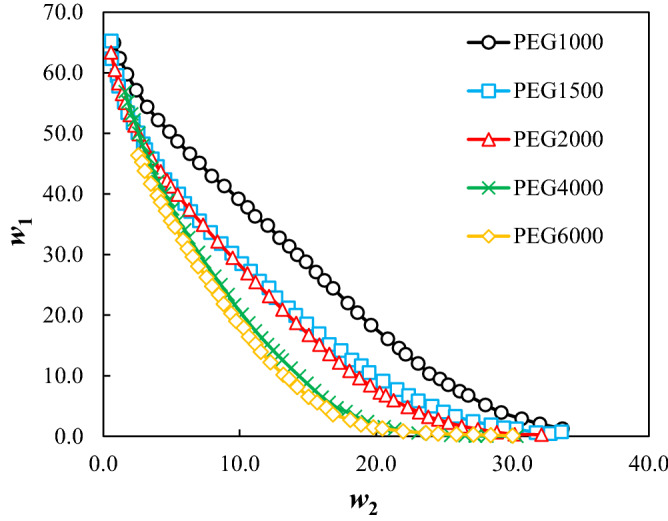
Effect of the molecular mass of PEG on binodal curves of PEG_1000,1500,2000,4000,6000_ (1) + trilithium citrate (2) + water (3) system at *T* = 298.15 K and *P* = 101 kPa.

The measured binodal curves of the ternary system composed of PEG_1000,1500,2000,4000,6000_ + trilithium citrate + water at *T* = 298.15 K were given in Table 2, and were shown in Fig. 1. The expansion of the biphasic area resulting from the increase of the MMP is obvious from Fig. 1. The mass fractions of the PEG and salt for the measured tie-lines were reported in Table 3. Moreover, the tie-line length (TLL) and slope (TLS) were computed using Eqs. (10) and (11):

10 $${\text{TLL}} = \left[ {(w_{1}^{{{\text{top}}}} - w_{1}^{{{\text{bot}}}} )^{2} + (w_{2}^{{{\text{top}}}} - w_{2}^{{{\text{bot}}}} )^{2} } \right]^{0.5}$$

11 $${\text{TLS = }}{{\left( {w_{1}^{{{\text{top}}}} - w_{1}^{{{\text{bot}}}} } \right)} \mathord{\left/ {\vphantom {{\left( {w_{1}^{{{\text{top}}}} - w_{1}^{{{\text{bot}}}} } \right)} {\left( {w_{2}^{{{\text{top}}}} - w_{2}^{{{\text{bot}}}} } \right)}}} \right. \kern-0pt} {\left( {w_{2}^{{{\text{top}}}} - w_{2}^{{{\text{bot}}}} } \right)}}$$

In these equations, 1 and 2 subscripts refer to the PEG and trilithium citrate, and, the “*top*” and “*bot*” superscripts show the polymer and salt-affluent regions, respectively. The estimated values of the TLL and TLS of the tie-lines were reported in Table 3. The raising of the biphasic area was clearly observed from TLL values; however, there is not a clear trend for TLS.

**Table 2:** Mass fractions of the PEG and trilithium citrate on binodal binodal curves of the studied ATPSs at *T* = 298.15 K and *P* = 101 KP.

100*w*_1_	100*w*_2_		100*w*_1_		100*w*_2_		100*w*_1_		100*w*_2_	
**PEG**_**1000**_** (*w***_**1**_**) + Trilithium citrate (*w***_**2**_**) + water**										
64.93	0.78		29.96		14.22		3.91		29.18	
62.39	1.20		28.79		14.87		2.88		30.68	
59.75	1.74		27.10		15.59		2.05		32.01	
57.11	2.42		25.73		16.22		1.25		33.67	
54.38	3.22		24.41		16.85					
52.19	4.02		21.99		17.93					
50.27	4.85		20.41		18.64					
48.68	5.44		18.33		19.63					
46.63	6.35		16.07		20.86					
45.11	7.05		14.54		21.69					
42.95	7.95		13.53		22.17					
41.31	8.87		12.02		23.09					
39.20	9.91		10.31		23.91					
37.79	10.58		9.45		24.69					
36.31	11.13		8.49		25.29					
34.79	12.06		7.43		26.14					
32.76	12.92		6.74		26.75					
31.37	13.63		5.16		28.02					
**PEG**_**1500**_** (*w***_**1**_**) + Trilithium citrate (*w***_**2**_**) + water**										
65.23	0.581		33.63		7.87		7.69		21.54	
62.31	0.631		31.81		8.65		6.71		22.39	
59.55	1.01		30.21		9.42		5.84		23.09	
57.77	1.12		28.50		10.15		4.95		24.10	
55.16	1.53		27.26		10.79		3.99		24.93	
53.4	1.79		25.61		11.45		3.29		25.81	
51.73	2.19		24.47		12.15		2.45		27.11	
50.08	2.55		22.87		12.65		1.89		28.43	
48.23	2.93		21.19		13.57		1.39		29.37	
47.29	3.11		19.95		14.10		1.20		30.29	
45.81	3.59		18.53		14.91		0.61		31.74	
44.53	3.97		16.93		15.82		0.44		32.77	
42.35	4.52		15.13		16.57		0.72		33.60	
41.26	4.97		14.14		17.43					
39.99	5.53		12.57		18.22					
38.43	5.98		11.64		18.92					
37.09	6.41		10.51		19.49					
35.57	7.05		9.10		20.51					
**PEG**_**2000**_** (*w***_**1**_**) + Trilithium citrate (*w***_**2**_**) + water**										
63.38	0.56		25.45		11.16		1.65		26.28	
60.48	0.84		23.13		12.15		1.19		27.46	
58.29	1.10		20.91		13.13		0.66		28.85	
56.51	1.36		18.71		14.14		0.33		30.16	
55.04	1.59		16.73		15.06		0.26		32.12	
53.03	1.93		15.13		15.84					
51.29	2.28		13.58		16.57					
49.86	2.59		12.17		17.29					
48.01	3.01		10.83		18.04					
45.88	3.54		9.58		18.77					
43.76	4.20		8.42		19.56					
42.26	4.67		7.21		20.27					
41.27	4.94		6.76		20.70					
39.91	5.44		5.96		21.28					
37.44	6.30		4.83		22.31					
34.91	7.31		3.96		23.15					
32.16	8.37		3.23		23.82					
**PEG**_**4000**_** (*w***_**1**_**) + Trilithium citrate (*w***_**2**_**) + water**										
56.89	1.60		25.01		8.62		3.29		18.49	
55.19	1.81		23.41		9.14		2.42		19.37	
53.23	2.08		21.72		9.62		1.21		21.36	
52.24	2.27		20.15		10.18		1.58		20.59	
50.03	2.60		18.74		10.66		0.55		23.08	
47.98	2.88		17.41		11.15		0.33		24.11	
46.04	3.28		16.25		11.61		0.16		25.47	
44.31	3.56		15.14		12.04		0.09		26.53	
44.29	3.56		14.17		12.45		0.06		27.26	
42.07	4.00		13.23		12.84		0.06		27.30	
40.11	4.49		12.59		13.13		0.03		28.12	
38.99	4.76		11.23		13.77		0.01		30.32	
37.54	5.07		9.96		14.38					
35.59	5.49		8.75		14.92					
34.09	5.97		7.56		15.49					
32.78	6.26		6.46		16.04					
30.36	6.97		5.59		16.55					
**PEG**_**6000**_** (*w***_**1**_**) + Trilithium citrate (*w***_**2**_**) + water**										
46.38		2.56		17.89		10.29		0.32		25.89
45.25		2.84		16.43		10.67		0.26		27.13
43.82		3.03		15.23		11.16		0.18		28.40
41.68		3.49		13.94		11.55		0.11		30.01
39.73		3.98		12.21		12.25				
38.63		4.22		10.16		13.20				
37.2		4.58		9.46		13.62				
35.56		4.96		8.15		14.22				
34.56		5.25		6.51		15.04				
32.35		5.84		5.59		15.68				
30.99		6.18		4.69		16.36				
29.63		6.55		3.62		16.84				
28.06		6.95		2.69		18.11				
26.24		7.57		1.95		18.89				
24.76		7.97		1.49		19.80				
23.35		8.47		1.29		20.46				
21.84		8.82		0.81		21.98				
20.27		9.38		0.54		23.64				
19.01		9.74		0.43		24.55				

Standard uncertainties, u, are u(*T*) = 0.1 K, u(*w*) = 0.0009, and u(*P*) = 5 kPa.

**Table 3:** Experimental tie-line mass factions (*w*) for PEG_1000,1500,2000,4000,6000_ (1) + trilithium citrate (2) + water (3) system at *T* = 298.15 K, and *P* = 101 KP, along with the tie-line length (TLL), and tie-line slop (TLS).

Stock solution		Top phase			Bott phase	− TLS	TLL	D_obs_	R	E%
100*w*_1_	100*w*_2_	100*w*_1_	100*w*_2_	100*w*_1_	100*w*_2_					
**PEG**_**1000**_** (*w***_**1**_**) + Trilithium citrate (*w***_**2**_**) + water**										
15.20	22.95	46.13	6.58	4.65	28.53	1.89	0.47	2.67	0.30	44.13
14.94	23.79	51.75	4.19	3.47	29.90	1.88	0.55	2.54	0.34	46.43
14.96	24.65	55.97	2.74	2.50	31.30	1.87	0.61	2.51	0.31	43.90
14.98	25.50	59.14	1.88	1.84	32.53	1.87	0.65	2.46	0.30	42.76
14.84	26.09	61.06	1.46	1.55	33.17	1.87	0.67	2.37	0.30	41.35
**PEG**_**1500**_** (*w***_**1**_**) + Trilithium citrate (*w***_**2**_**) + water**										
15.02	17.94	35.25	7.19	6.42	22.51	1.88	0.33	1.79	0.42	43.20
14.97	19.28	43.64	4.16	3.73	25.21	1.9	0.45	1.82	0.39	41.63
14.95	20.64	51.12	2.27	2.47	26.97	1.97	0.55	1.64	0.34	36.12
14.10	21.89	55.76	1.45	1.98	27.83	2.04	0.60	1.47	0.31	31.62
14.99	21.9	57.23	1.24	1.71	28.40	2.05	0.62	1.48	0.29	30.09
14.90	24.39	64.78	0.46	0.75	31.18	2.09	0.71	1.83	0.28	34.16
**PEG**_**2000**_** (*w***_**1**_**) + Trilithium citrate (*w***_**2**_**) + water**										
15.57	17.34	35.70	6.98	3.50	23.56	1.94	0.36	1.12	0.60	40.18
15.57	19.17	44.8	3.86	1.53	26.52	1.91	0.49	1.15	0.48	35.59
15.10	20.55	51.57	2.21	1.08	27.60	1.99	0.57	1.18	0.38	31.21
15.56	21.82	53.12	1.91	0.49	29.80	1.89	0.60	1.11	0.40	30.81
15.57	24.38	57.24	1.25	0.13	32.96	1.80	0.65	1.21	0.37	30.96
**PEG**_**4000**_** (*w***_**1**_**) + Trilithium citrate (*w***_**2**_**) + water**										
14.91	15.72	41.67	4.10	1.00	21.76	2.30	0.44	1.06	0.52	35.53
14.75	17.09	43.59	3.70	0.41	23.74	2.15	0.48	1.04	0.50	34.09
14.96	19.05	47.51	2.96	0.10	26.40	2.02	0.53	0.91	0.46	29.35
15.41	20.47	52.66	2.17	0.04	28.02	2.04	0.59	0.81	0.41	25.05
14.88	21.89	56.01	1.74	0.02	29.17	2.04	0.62	1.01	0.36	26.74
**PEG**_**6000**_** (*w***_**1**_**) + Trilithium citrate (*w***_**2**_**) + water**										
25.04	9.15	33.37	5.49	1.45	19.52	2.28	0.35	0.50	5.19	72.18
25.04	10.48	36.43	4.71	0.27	23.06	1.97	0.41	0.47	2.83	57.09
25.01	11.57	38.46	4.22	0.07	25.21	1.83	0.44	0.48	2.18	51.09
25.02	12.78	42.03	3.44	0.03	26.51	1.82	0.48	0.49	1.85	47.61
25.01	13.82	45.05	2.86	0.01	27.51	1.83	0.51	0.55	1.47	44.68
25.06	15.05	47.96	2.36	0.00	28.93	1.81	0.55	0.47	1.25	36.97

The measured partitioning coefficient (*D*_*obs*_), the extraction efficiency percent (*E*%), and the separation coefficient (*R*) of the salbutamol in the studied tie-lines were also reported.

Standard uncertainties, u, are u(*T*) = 0.1 K, u(*w*) = 0.0013, and u(*P*) = 5 kPa.

**Figure 2:**
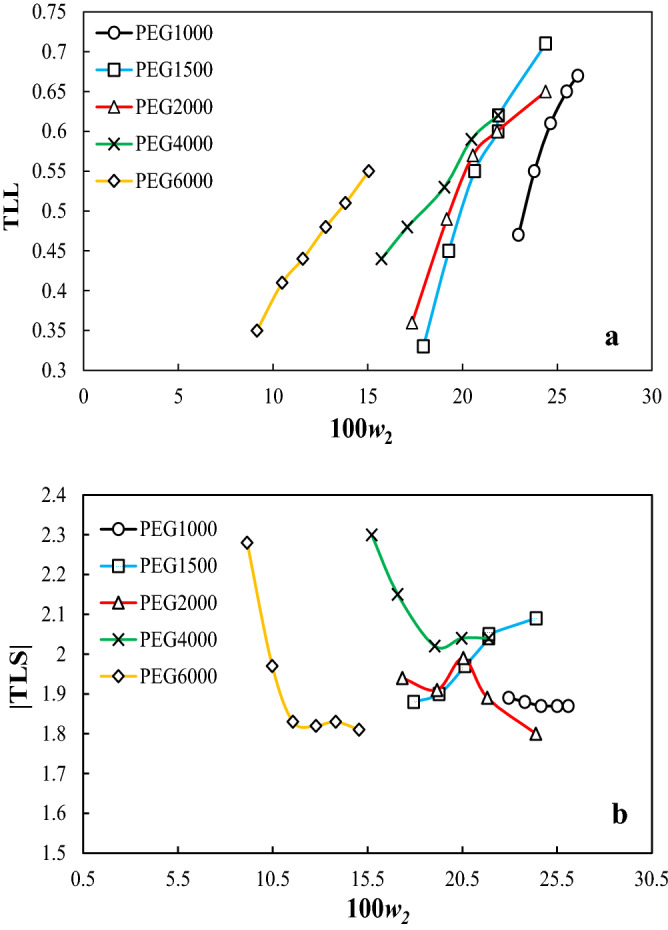
(**a**) The tie-line length (*TLL*) as a function of the mass fraction of the salt in the stock solution (*w*_2_); (**b**) the slope of the tie lines (|TLS|) as a function of the mass of the salt in the stock solution (*w*_2_) for PEG_1000,1500,2000,4000,6000_ (1) + trilithium citrate (2) + water (3) two-phase system at *T* = 298.15 K.

The TLL and TLS of the studied tie-line were graphically shown as a function of the mass fraction of the salt in stock-solution in Fig. 2a, and b, respectively. It is obvious from Fig. 2a that, as the MMP was increased, the TLL was increased, which confirms that the surface area of the biphasic area was enlarged.

In other words, by increasing MMP, and therefore the chain length of the polymer, the hydrophobic interactions between the polymer chains were increased, and therefore, the tendency of the polymer chains to separate from the aqueous solution as an independent phase was increased. So, the tendency of the PEG + trilithium + water system to form a biphasic system was increased.

### Correlation of the binodal and tie-line composition

One of the major parts of any thermodynamics study is the modeling of the measured data. In fact, a desirable model can provide a mathematical description of the experimental results, which can be used in further studies more precisely. So, several acceptable models have been applied in this work to correlate the experimental tie-line data, Indeed, Setschenow^18^, osmotic virial^42^, and the electrolyte-Wilson^39^ models were applied to model the thermodynamics of the studied ATPSs.

The experimental binodal curves were fitted with a 3-parameter non-linear Merchuk equation^25^:

12 $$w_{1} = a\exp (bw_{2}^{0.5} - cw_{2}^{3} )$$

The parameters of this equation (i.e., *a*, *b*, and *c*) were obtained from the non-linear least-square fitting of the mass fraction of PEG (*w*_1_) and trilithium citrate (*w*_2_).

The experimental binodal data were fitted with Eq. (12) by minimizing the least squares errors of the following objective function (OF),

13 $$OF\left( {a,b,c} \right) = \sum\limits_{i} {\left\{ {\left[ {a\exp \left( {bw_{2}^{0.5} - cw_{2}^{3} } \right)} \right] - w_{1,\exp ,i} } \right\}^{2} }$$

In which, *w*_1_, _*exp*, *i*_ is the *i'*th experimental mass fraction of the PEG at desired binodal curve. In minimization of Eq. (13) the nonlinear Levenberg–Marquardt optimization algorithm was used. The fitting results, together with the statical analysis of the goodness of fit (i.e., average absolute deviation (AAD%), standard deviation SD, and the coefficient of determination R^2^) were presented in Table 4. The adequate consistency between the correlated the measured binodal data can be inferred from the results reported in Table 4.

**Table 4:** Values of parameters of Eq. 12 for PEG_1000,1500,2000,4000,6000_ (1) + trilithium citrate (2) + water (3) system at *T* = 298.15 K.

	MM_*PEG*_	*a*	*b*	10^−2^*c*	100SD	100AAD
Equation (12)	1000	0.7687	− 1.9063	0.769	10.8729	7.9204
	1500	0.7867	− 2.8535	1.0091	19.4625	15.3267
	2000	0.7829	− 2.8003	1.337	6.3627	5.7621
	4000	0.9451	− 3.955	2.6249	14.9469	10.8386
	6000	0.8759	− 3.8895	3.2074	21.513	19.2917

${\text{AAD\% }} = \sum\nolimits_{j = 1}^{N} {{{100\left| {w_{j}^{exp} - w_{j}^{cal} } \right|} \mathord{\left/ {\vphantom {{100\left| {w_{j}^{exp} - w_{j}^{cal} } \right|} N}} \right. \kern-0pt} N}}$, and ${\text{SD\% }} = \sum\nolimits_{j = 1}^{N} {{{100\left( {w_{j}^{exp} - w_{j}^{cal} } \right)^{2} } \mathord{\left/ {\vphantom {{100\left( {w_{j}^{exp} - w_{j}^{cal} } \right)^{2} } N}} \right. \kern-0pt} N}}$. AAD% and SD% are the average absolute deviation percent and standard deviation percent between the experimental (*exp*) and calculated (*cal*) values, and *w* is the mass fraction of the PEG_1000,1500,2000,4000,6000_. Also, *N* is the number of the points in the binodal curves.

The solute–solvent interactions may change the structure of the solvent. The hydrogen-bonding between the solute and water is the principal interaction impress the solvent structure. Some solutes can increase the intermolecular interaction between the water molecules, and therefore, water molecules are aligned next to each other in a more regular structure; these solutes are kosmotropic salts^43^. The dissolution of some other solutes may also result in a disordered hydrogen-bonding structure of the water network. The second class of the solutes are Chaotropic salts^44^. Moreover, the dissolution of kosmotropic salts in aqueous solution at a critical concentration can salting-out the other compounds form the aqueous solution.

The salting-out capability of PEG + salt + water ATPS has been investigated in the present work using the Setschenow-type model^13,45^:

14 $$\ln \left( {\frac{{C_{1}^{top} }}{{C_{1}^{bot} }}} \right) = k_{0} + k_{s} \left( {C_{2}^{bot} - C_{2}^{top} } \right)$$

where *k*_*s*_ and *k*_0_ are fitting parameters, and the *C*_1_ and *C*_2_ refers to the molarity of PEG and tri-lithium citrate, respectively. Moreover, *top* and *bot* superscripts refer to the PEG and trilithium citrate-affluent regions, respectively. Furthermore, *k*_*s*_ is a constant repeatedly used in the literature^13^ to quantify the salting-out capability of the ATPSs. So that, the more the *k*_*s*_ the more the two-phase formation tendency of the ATPS. Calculated *k*_*s*_ values were reported in Table 5. The enhancement of the salting-out coefficient, *k*_*s*_, with increasing the MMP, and the salting-out ability of the studied ATPSs is obvious from the Table 5.

**Table 5:** Results of the fitting of the experimental tie-line compositions with the Seteschenow-type model (i.e., Eq. 14) for PEG_1000,1500,2000,4000,6000_ (1) + trilithium citrate (2) + water (3) system at *T* = 298.15 K.

*MMP*_*PEG*_	*k*_0_	*k*_s_	100DEV	R^2^
1000	0.1139	1.8175	1.1724	0.9966
1500	− 0.6161	2.6255	3.4685	0.9873
2000	− 0.6768	3.2027	5.8175	0.9873
4000	− 3.0357	6.8817	1.4139	0.9507
6000	− 2.7436	7.6304	1.9655	0.9646

R^2^ is the coefficient of determination for linear relation of $\ln \left( {{{C_{1}^{top} } \mathord{\left/ {\vphantom {{C_{1}^{top} } {C_{1}^{bot} }}} \right. \kern-0pt} {C_{1}^{bot} }}} \right)$ with $\left( {C_{2}^{bot} - C_{2}^{top} } \right)$. As well, $DEV\% = \sum\nolimits_{p,l.j} {{{100\left( {w_{p,l,j}^{\exp } - w_{p,l,j}^{cal} } \right)} \mathord{\left/ {\vphantom {{100\left( {w_{p,l,j}^{\exp } - w_{p,l,j}^{cal} } \right)} {6N}}} \right. \kern-0pt} {6N}}}$, where *w*_*p,l,j*_ is the mass fraction of component *j* (i.e. polymer, salt, or water) for *l*th tie-line composition in the phase *p*, and *N* is the number of experimental (*exp*) or calculated(*cal*) tie-line compositions.

Liquid–liquid equilibria of PEG_1000,1500,2000,4000,6000_ + trilithium citrate + water was also modeled using the virial expansion of the chemical potential (*µ*)^42^. The following relations were applied to estimate the *µ* of the PEG (*µ*_1_) and trilithium citrate (*µ*_2_):

15 $$\mu_{1} = \mu_{1}^{ \circ } + RT\left( {\ln m_{1} + \beta_{11} m_{1} + \beta_{12} m_{2} } \right)$$

16 $$\mu_{2} = \mu_{2}^{ \circ } + RT\left( {\ln m_{2} + \beta_{22} m_{2} + \beta_{12} m_{1} } \right)$$

In these relations, the standard state of *µ* displayed as $\mu_{i}^{ \circ } ,i = 1,2$, whereas, 1 and 2 subscripts refer to the PEG and electrolyte. Also, *β*_*ij*_ represented the interaction of *i* and *j* component. The chemical potential of water (*µ*_3_) can also be derived from the Gibbs–Duhem relation as follows:

17 $$\mu_{3} = \mu_{3}^{ \circ } - RTV_{3} \rho \left( {m_{1} + m_{2} + \frac{{\beta_{11} }}{2}m_{1}^{2} + \frac{{\beta_{22} }}{2}m_{2}^{2} + \beta_{12} m_{1} m_{2} } \right)$$

The fitting parameters (i.e., *β*_*ij*_ with *i*, *j* = 1, 2, and 3) were obtained by minimizing the following objective function, and are reported in Table 6.

18 $$OF = \sum\nolimits_{p,l,j} {100(w_{p,l,j}^{exp} - w_{p,l,j}^{cal} )^{2} }$$

In Eq. (18), subscribes of “*exp*” and “*cal*” are experimental and calculated mass fractions, respectively; *p* indicates a phase (top-phase or bottom), of *l’*th tie-line, and *j* refers toe the PEG, salt, or water. Also, the condition of equal chemical potential for all components (i.e., $\mu_{i}^{top} = \mu_{i}^{bot}$, where *i* = 1, 2 and 3) has been applied to establish the thermodynamic equilibria, and to correlate the tie-line data with the model^46^.

**Table 6:** Results of the correlation of the experimental tie-line compositions using the osmotic virial equation for PEG_1000,1500,2000,4000,6000_ (1) + trilithium citrate (2) + water (3) system at *T* = 298.15 K.

*M*_*PEG*_	*β*_11_	*β*_12_	*β*_22_	100DEV
1000	1.2906	2.7838	1.0427	19.9900
1500	− 1.5422	1.5204	− 0.4539	11.5130
2000	3.2112	3.6025	0.0013	22.263
4000	− 34.9002	− 1.2723	− 1.5677	27.208
6000	30.6107	8.4560	− 0.4381	24.616

$${\text{DEV\% }} = {{\sum\nolimits_{p,l,j} {100\left( {w_{p,l,j}^{exp} - w_{p,l,j}^{cal} } \right)^{2} } } \mathord{\left/ {\vphantom {{\sum\nolimits_{p,l,j} {100\left( {w_{p,l,j}^{exp} - w_{p,l,j}^{cal} } \right)^{2} } } {6N}}} \right. \kern-0pt} {6N}}$$.

**Figures 3:**
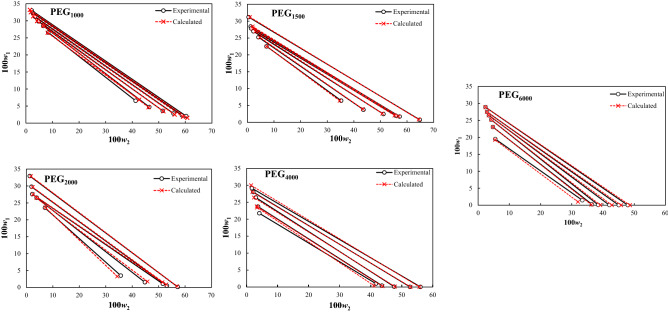
Comparison of the experimental and calculated tie-line compositions using osmotic virial model at *T* = 298.15 K.

The experimental compositions of the measured tie-line lines are compared with the calculated concentration from the osmotic virial model in Fig. 3. The estimated parameters of the model were also reported in Table 6. An acceptable fitting results can be inferred from Fig. 3 and Table 6.

**Figures 4:**
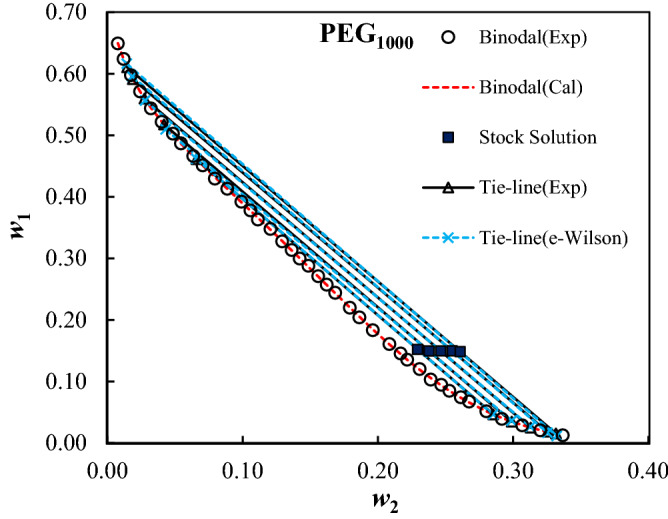
Experimental (*Exp*) and calculated (*Cal*) binodal curves using Merchuk equation, and tie-line composition using e-Wilson model for PEG_1000_ (1) + trilithium citrate (2) + water (3) ATPSs at *T* = 298.15 K, and *P* = 101 kPa.

**Figures 5:**
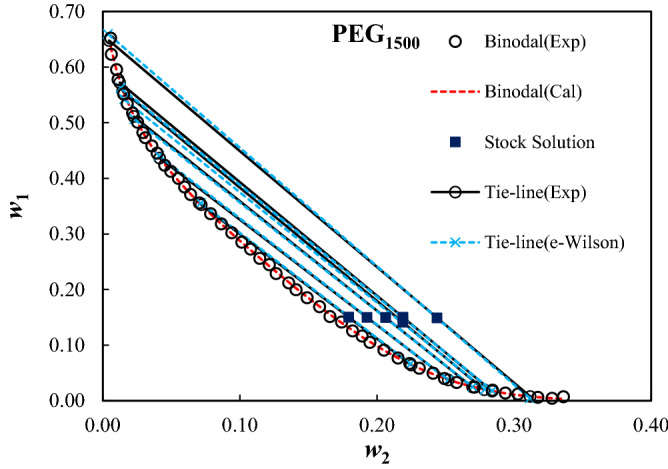
Like Fig. 5 for PEG_1500_(1) + trilithium citrate (2) + water (3) ATPSs.

**Figures 6:**
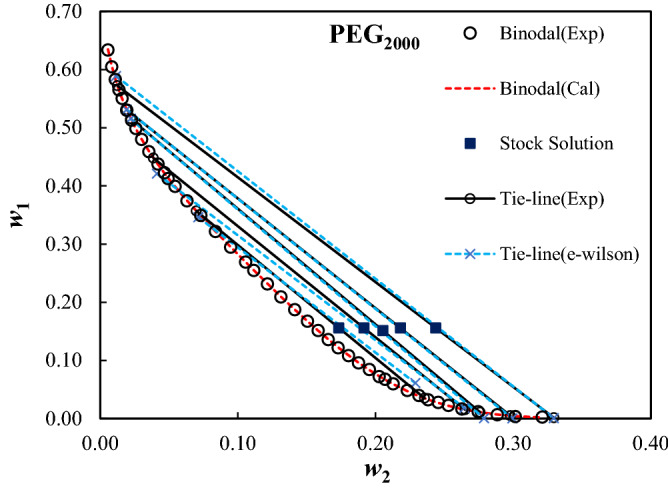
Like Fig. 5 for PEG_2000_(1) + trilithium citrate (2) + water (3) ATPSs.

**Figures 7:**
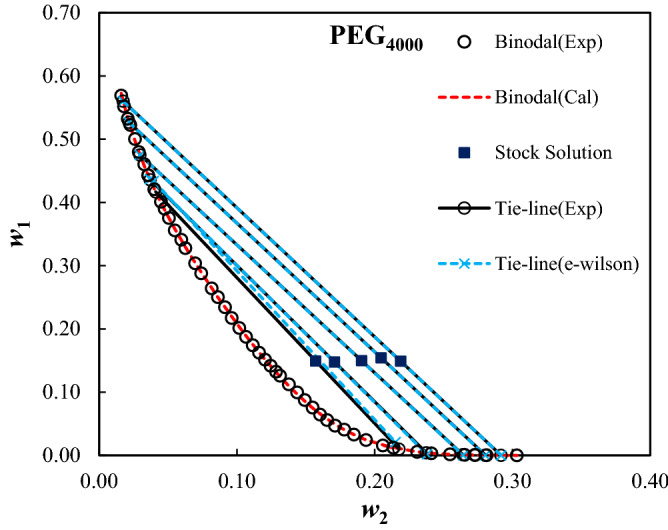
Like Fig. 5 for PEG_4000_(1) + trilithium citrate (2) + water (3) ATPSs.

**Figures 8:**
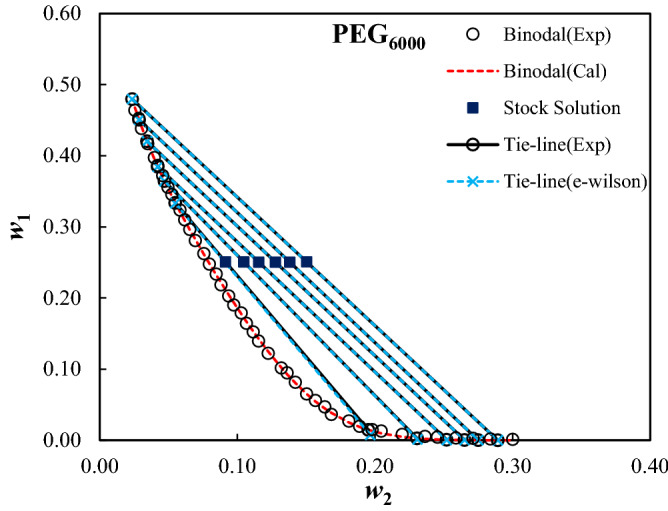
Like Fig. 5 for PEG_6000_(1) + trilithium citrate (2) + water (3) ATPSs.

**Figures 9:**
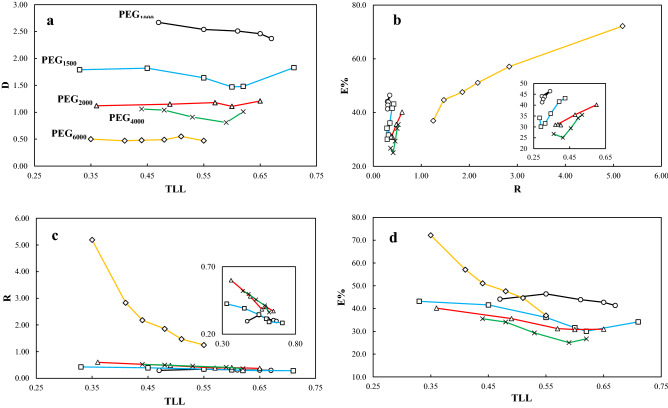
(**a**) Partitioning coefficient (*D*) as a function of tie-line length (*TLL*); (**b**) Extraction efficiency percent (*E*%) as a function of separation coefficient (*R*); (**c**) *R* as a function of TLL; (**d**) *E*% as a function of TLL; for partitioning of salbutamol in the top and bottom phases of PEG_1000,1500,2000,4000,6000_ (1) + trilithium citrate (2) + water (3) at *T* = 298.15 K, and *P* = 101 kPa. In these figures, (○) is for ATPSs of PEG_1000_, (□)PEG_1500_, (∆)PEG_2000_, ( ×) PEG_4000_, and (◊) PEG_6000_.

The hydrophobic interactions and the solvent can control the properties like virial coefficients and volume of solute in solution phase. Zafarani-Moattar et al.^47^ showed that the infinite dilution apparent molal volume ($V_{\varphi ,m}^{\infty }$) of PEG is independent of the molecular mass of the polymer, and the value of $V_{\varphi ,m}^{\infty } \simeq 37.0$ cm^3^/mol was reported for PEG_2000_ and PEG_4000_ at *T* = 298.15 K. The limiting $V_{\varphi ,m}^{\infty } = 149.35$ at *T* = 298.15 K for trilithium citrate in aqueous solution was reported by Devi and coworkers^48^. However, they also found that, the $V_{\varphi ,m}^{\infty }$ values were decreased in aqueous solution by increasing the concentration of the [Emim][HSO_4_] ionic liquid. They also concluded that, the hydrophobic-hydrophobic and ion-hydrophobic interactions are more dominant than the hydrophilic-hydrophilic and ion-hydrophilic interactions in aqueous solutions. The increasing behavior of the polymer–polymer (*β*_11_) and polymer-salt (*β*_12_) virial coefficients with increasing the molecular mass of polymer (MMP) can be seen from Table 6, which is more obvious for *β*_11_. Whereas, the salt-salt (*β*_22_) virial coefficient is almost independent of molecular mass of PEG. In other words, it seems that, by increasing the MMP, especially in the case of PEG_4000_ and PEG_6000_, the polymer–polymer interactions were increased more substantially than the PEG-salt interactions, which motivated the PEG particles to exclude from the rest of the solution as an independent phase. In other words, this simple model provides a microscopic view of the molecular interaction. By increasing the MMP, and therefore, increasing the number of the monomers of the PEG, the probability of monomer–monomer interactions was more likely, compared to the water-monomer and ion-monomer interactions. Therefore, the system is more favorable to form a biphasic ATPS. This conclusion agrees with the experimental results of this work, whereas, the two-phase formation affinity of the studied ATPSs were increased by increasing the molecular mass of PEG.

Electrolyte-Wilson model was extended for the aqueous systems of polymer + electrolyte + water systems by Sadeghi et al.^39^ All equations, definitions and information about chemical potential, activity coefficients and phase equilibrium condition of this model were demonstrated in our previous works^13,41^, and only the results of  the tie-line correlation for ATPSs composed of PEG_1000,1500,2000,4000,6000_ + trilithium citrate + water of data were reported in this work. Furthermore, the dielectric constant, *D*_*s*_, molar volume, *V*_*s*_, and the estimated number of the segments for each polymer (i.e., *r*_1_) were reported in Table 7.

**Table 7:** Dielectric constant (*D*_*s*_) and molar volume (*V*_*s*_) of different molecular mass of PEG_1000,1500,2000,4000,6000_ (1) and water (3) at desired temperature in modeling of the e-Wilson model.

*MM*_*PEG*_	*D*_*s*,1_	*D*_*s*,3_ (m^3^/kg)	10^5^ *V*_*s*,3_	10^−6^ *V*_s,1_	r_1_
1000	2.2			841.7	46.5832
1500	2.2			845	70.1488
2000	2.2	997.045	1.8068735	1877	103.88
4000	2.2			3551	196.53
6000	2.2			83,161	276.15

Dielectric constant (D_s_) were given from Madelung et al.^49^, Also, Molar volume of PEG_1000_,_1500_ were obtained from^50,51^, for PEG_2000,4000_ were from^47^, and for PEG_6000_ was from^52^. The molar volume of water was calculated from the equation presented by Robinson and Stocks^53^.

The calculated parameters of the e-Wilson model were reported in Table 8, and the experimental and correlated results were compared in Figs. 4, 5, 6, 7 and 8. The excellent concurrence of the modeled and experimental tie-lines is obvious from Table 8 and Figs. 5, 6, 7 and 8 for all cases. Therefore, e-Wilson model can fit the tie-lines compositions, satisfactorily.

**Table 8:** Results of the modelling of the experimental tie-line compositions with e-Wilson model for PEG_1000,1500,2000,4000,6000_ (1) + trilithium citrate (2) + water (3) system at *T* = 298.15 K.

*M*_*PEG*_	*H*_1*.*2_	*H*_2*.*1_	*H*_3,2_	*H*_2,3_	*H*_1,3_	*H*_3,1_	DEV%
1000	− 0.7147	0.4898	0.6365	0.6631	− 1.2372	0.3364	0.2304
1500	− 0.4289	0.2453	0.6723	0.3799	− 1.4913	0.2378	0.2793
2000	0.0601	0.4234	0.0193	7.7171	0.2585	0.2576	1.1183
4000	− 0.0092	− 3.2099	0.0191	− 7.5011	3.9599	0.1195	0.2106
6000	− 0.0488	14.6031	0.0323	6.9646	0.9022	1.7227	0.0246

### The effect of molecular mass of PEG on salbutamol partitioning

Partitioning of salbutamol in tie-line solutions were measured at *T* = 298.15 K, and the pressure of *p* = 1.0 atm. The values of the partitioning coefficient, *D*, separation coefficient, *R*, and extraction efficiency percent, *E*%, were reported in Table 3, and were shown graphically in Fig. 9.

Different MMPs (i.e., MMP = 1000, 1500, 2000, 4000, and 6000) were used in the investigated ATPS for salbutamol partitioning. According to Fig. 9a, the salbutamol concentration in the polymer-affluent phase was superior than the bottom phase for PEG_1000_ and PEG_1500_. So that, the maximum value of *D* = 2.67 was observed in PEG_1000_. This value is approximately equal in both phases for PEG_2000_ and PEG_4000_, and the average of the *D* is 1.15 and 0.96 for PEG_2000_, and PEG PEG_4000_, respectively. In PEG_6000_, the drug was concentrated in the salt-affluent phase, i.e., *D* is ~ 0.5. Therefore, at *T* = 298.15 K the lower MMP of PEG resulted in the higher partitioning coefficient, and polymers with higher molecular mass have more acceptable outcomes for drug extraction in aqueous solvents. Also, the standard deviations of the obtained *D* values are 0.1, 0.15, 0.04, 0.09, and 0.03 for ATPSs composed of PEGs with MMP = 1000, 1500, 2000, 4000, and 6000, respectively, which, confirm that *D* is independent of TLL. More precisely, only in the case of PEG_1000_, the *D* was slightly decreased by increasing the TLL, and inversely, for PEG_2000_ an almost increasing behavior of *D* with an increase of the TLL was observed.

The efficiency (*E*%) as function of separation coefficient, *R*, and TLL were shown in Fig. 9b, and d, respectively. In all cases, other than PEG_6000_, the *E*% values are lower than 50%, and the efficiency was decreased by increasing the MMP until 4000. The separation ability as function of TLL, as shown in Fig. 9c, also demonstrate that, other than PEG_6000_, the R values are lower than 1.0, which is almost increased by increasing the MMP.

The inconsistence behavior of PEG_6000_ is due to the more tendency of PEG_6000_ to form a biphasic system. Indeed, from Fig. 9c it can be inferred that, the mass of the polymer-affluent phase is higher than the salt-affluent phase. In other words, PEG_6000_ can catch more water molecules from the stock solution in competition with the ionic components, compared to the other polymers. However, in all other cases the mass of salt-affluent phase is more than the top-phase, due to the more water molecules cached with the ionic components when contest with the polymer chains.

## Conclusion

In this paper, LLE phase diagrams for the systems of PEG_1000,1500,2000,4000,6000_ + trilithium citrate + water at *T* = 298.15 K were reported. The binodal curves were collected and fitted susccesfully by the Merchuk equation. Setschenow-type model were applied to study the salting-out capability. It was found that, salting-out parameter (*k*_*s*_) was boosted with enhancement of MMP. In other words, the tendency of the salt-affluent phase was intensified to adopt the H_2_O moleculs with increasing the length of the polymer chain. Furthermore, osmotic virial and electrolyte-Wilson models were implemented for thermodynamical modeling of the phase equilibrium. It was found that, there is a direct relationship between the MMP, binodal curves extent, and the tie-line length. So that, the more the MMP the more the two-phase extent of the phase diagram. Besides, partitioning coefficient (*D*) of salbutamol was measured in the studied ATPSs. According to the obtained data, it can be claimed that, the partioning coeffeicnet (*D*) of salbutamol was decreased with increasing the MMP. PEG_6000_ with minimum amount of *D* = 0.47, and high efficiency parcentage of 72% was appropriate for drug dissolution in the salt-affluent phase, and PEG_1000_ is better to transfer the salbutamol molecules to the polymer-affluent phase. Therfore, partitioning of salbutamol can be controlled by changing the MMP in PEG + trilithium citrate + water ATPS.

## Data Availability

All data generated or analyzed during this study are included in this published article.
